# Manure Microbial Communities and Resistance Profiles Reconfigure after Transition to Manure Pits and Differ from Those in Fertilized Field Soil

**DOI:** 10.1128/mBio.00798-21

**Published:** 2021-05-11

**Authors:** Kimberley V. Sukhum, Rhiannon C. Vargas, Manish Boolchandani, Alaric W. D’Souza, Sanket Patel, Akhil Kesaraju, Gretchen Walljasper, Harshad Hegde, Zhan Ye, Robert K. Valenzuela, Paul Gunderson, Casper Bendixsen, Gautam Dantas, Sanjay K. Shukla

**Affiliations:** a The Edison Family Center for Genome Sciences and Systems Biology, Washington University School of Medicine in St. Louis, St. Louis, Missouri, USA; b Department of Pathology and Immunology, Washington University School of Medicine in St. Louis, St. Louis, Missouri, USA; c Center for Oral Systemic Health, Marshfield Clinic Research Institute, Marshfield, Wisconsin, USA; d Center for Precision Medicine Research, Marshfield Clinic Research Institute, Marshfield, Wisconsin, USA; e Lake Region State College, Devils Lake, North Dakota, USA; f National Farm Medicine Center, Marshfield Clinic Research Institute, Marshfield, Wisconsin, USA; g Department of Molecular Microbiology, Washington University School of Medicine in St. Louis, St. Louis, Missouri, USA; h Department of Biomedical Engineering, Washington University in St. Louis, St. Louis, Missouri, USA; i Computation and Informatics in Biology Program, University of Wisconsin, Madison, Wisconsin, USA; University of British Columbia

**Keywords:** agriculture, antimicrobial resistance, dairy farm, manure, microbiome

## Abstract

In agricultural settings, microbes and antimicrobial resistance genes (ARGs) have the potential to be transferred across diverse environments and ecosystems. The consequences of these microbial transfers are unclear and understudied. On dairy farms, the storage of cow manure in manure pits and subsequent application to field soil as a fertilizer may facilitate the spread of the mammalian gut microbiome and its associated ARGs to the environment. To determine the extent of both taxonomic and resistance similarity during these transitions, we collected fresh manure, manure from pits, and field soil across 15 different dairy farms for three consecutive seasons. We used a combination of shotgun metagenomic sequencing and functional metagenomics to quantitatively interrogate taxonomic and ARG compositional variation on farms. We found that as the microbiome transitions from fresh dairy cow manure to manure pits, microbial taxonomic compositions and resistance profiles experience distinct restructuring, including decreases in alpha diversity and shifts in specific ARG abundances that potentially correspond to fresh manure going from a gut-structured community to an environment-structured community. Further, we did not find evidence of shared microbial community or a transfer of ARGs between manure and field soil microbiomes. Our results suggest that fresh manure experiences a compositional change in manure pits during storage and that the storage of manure in manure pits does not result in a depletion of ARGs. We did not find evidence of taxonomic or ARG restructuring of soil microbiota with the application of manure to field soils, as soil communities remained resilient to manure-induced perturbation.

## INTRODUCTION

In agricultural environments, microbes and the genes they carry have the potential to be exchanged and transferred by agricultural activity across large environmental settings. For example, on dairy farms, cows excrete fresh manure, which is stored in manure pits for 6 months to up to a year and then spread across agricultural land to promote crop growth and yield. This is of potential local and global concern for three reasons: (i) the restructuring of the microbiome of fresh manure in manure pits during storage could result in the emergence of potential pathobionts, (ii) agricultural practices could result in the spread of mammalian-associated pathobionts into atypical environments, and (iii) domesticated and food-producing animals receive antimicrobials that can overlap those used in humans to treat infections, which could result in the spread and transfer of antimicrobial resistance to environmental microbes (1, 2). However, our understanding of the microbiome consequences of these exchanges is limited (3). While many studies have aimed at better surveying and tracking mammal-based antimicrobial resistance spread in agricultural settings, no study has examined the interplay of microbial community and resistance correlations across the three environments (fresh manure, manure pits, and field soils) (1, 2, 4–12).

Antimicrobial use in food-producing animals is dominated by cattle, and the four primary drug classes used are tetracyclines, aminoglycosides, macrolides, and sulfa drugs (13). Antimicrobial usage can result in compositional shifts in manure in different ways. First, antimicrobials are not fully metabolized *in vivo*, and upwards of 70 to 90% may be excreted in manure and urine (14–16). These excreted antimicrobials could directly impact microbiome communities into which they are introduced, such as those in manure pits (17, 18). Second, antimicrobial usage could lead to the maintenance and evolution of antimicrobial resistant strains and genes in the gastrointestinal tract of domesticated animals, which could then be excreted (19–21). In both cases, we expect the microbiome and resistome composition of manure to be impacted by antimicrobial use. Further, the excrement can then be stored in manure pits and used as a source of nutrients across agricultural fields, allowing an opportunity for the spread of the microbiota of the manure to soils (22–24).

Manure provides essential nutrients for crop growth, such as phosphorus, potassium, and nitrogen, and adding organic matter to soils can improve soil structure and increase the soil’s ability to hold water and nutrients (25). Soil has a very different microbial community than the mammalian gut and can also harbor antimicrobial resistance genes (ARGs) (26–28). The agricultural soil microbiome is highly diverse, and naturally occurring ARGs are not necessarily related to antimicrobials used to treat animals (29). However, the introduction of microbiota from the guts of animals may have adverse effects on the microbial composition and functional resistance profiles found in the environment. Past studies suggest that antimicrobial resistance may remain on farms through the acquisition of multidrug resistance and horizontal gene transfer, but it is unclear how much antimicrobial resistance spreads to fields and how that spread influences microbial composition and the total amount and diversity of functional ARGs, known as the resistome of manure and soil (30–32). Further, most studies focus on known or individual antimicrobial resistance genes of interest, missing less characterized antimicrobial genes that may be of equal concern (7–12). Comprehensive functional metagenomic studies show that many ARGs are diverse, widespread, and increasingly novel (29, 33–35). It is reported that functional metagenomic selections identify genes with less than 65% amino acid identity to known resistance genes (35). As a result, the ARG profiles in dairy farms are yet to be comprehensively characterized.

The microbiome of soil is rich, complex, and variable by soil depth (36). This variation is influenced by physical (pH, oxygen availability, and water penetration) and biological (microbes, plants, and animals) differences in these environments (37, 38). The influence of the manure microbiome on soil samples could be greater at shallower soil depths, where it is initially spread, than at lower depths, where it may not easily penetrate. Further, the physical and chemical differences that exist in manure pits, which are generally stagnant, likely result in increasingly anaerobic environments deeper in the pit, in turn enriching for anaerobic microbes along a depth gradient.

**FIG 1:**
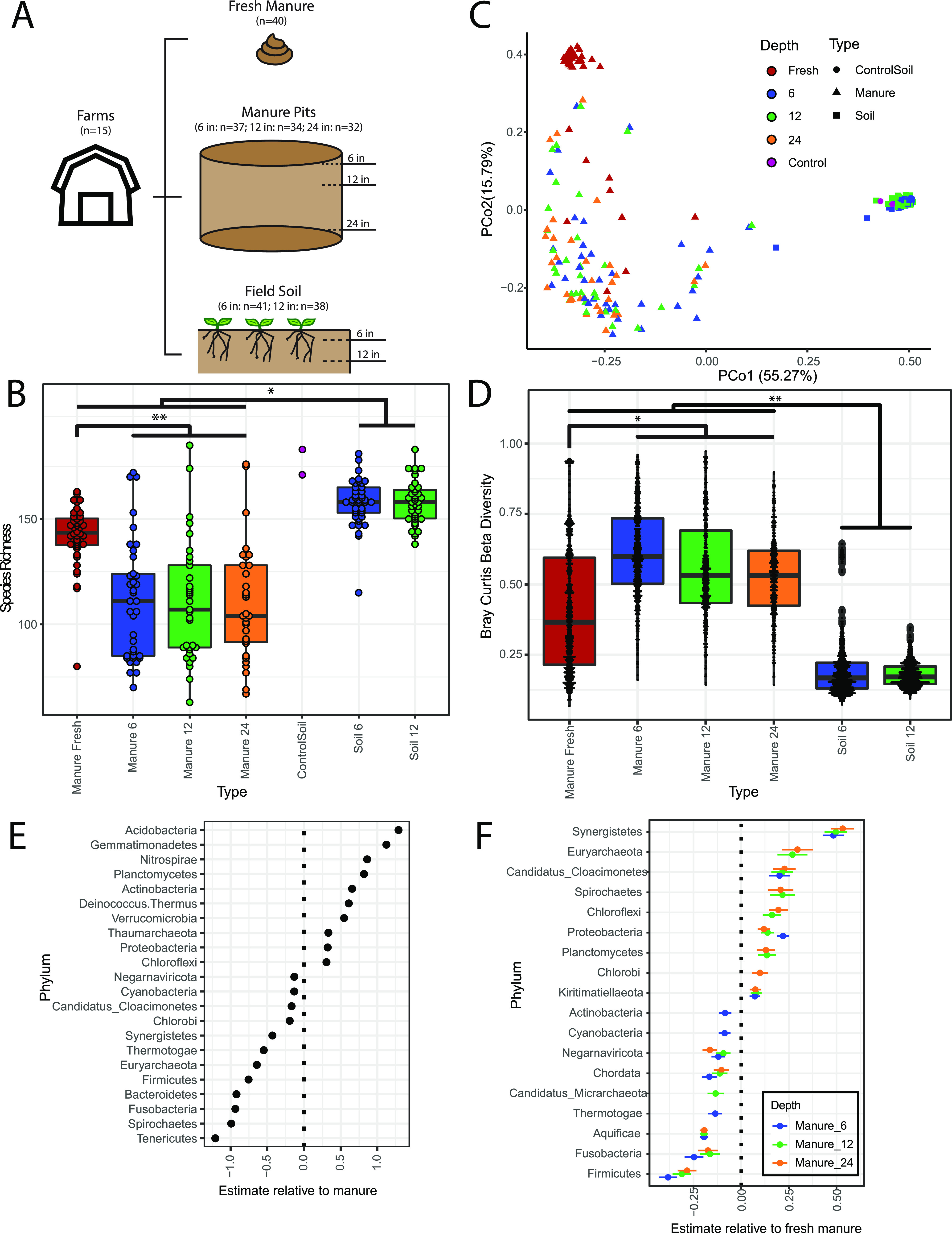
Taxonomic diversity metrics varied across fresh manure, manure pit, and soil samples. (A) Overview of study design and sample types. Samples were collected from 15 different farms. Manure pits were sampled at 3 depths (6 in., 12 in., and 24 in.). Field soil was sampled at two depths (6 in. and 12 in.). (B) Box plot of species richness by sample type. Points indicate each individual sample measured. Significance was determined by a linear mixed-effects model with random effects as location of sampling (marginal *R*^2^ = 0.516, conditional *R*^2^ = 0.584), followed by least-square means pairwise comparisons. Fresh manure was significantly different from manure pit samples (*P* < 0.001). Soil samples were significantly different from manure samples (*P* < 0.05). (C) Principal-coordinate analysis (PCoA) plot of Bray-Curtis dissimilarity index for species abundances of all sample types. There is significant clustering by sample type after controlling for repeated measures of sampling location (PERMANOVA, *R*^2^ = 0.45, *P* < 0.001). (D) Box plot of beta diversity determined by Bray-Curtis dissimilarity comparisons for each sample time. Points indicate pairwise comparisons by sample type. (E) Estimates of coefficients of soil relative to manure for significant phyla in a multivariable general linear model using MaAsLin2. Random effects included farm sample location and sampling time period. (F) Estimates of coefficients of manure pit depths relative to fresh manure for significant phyla in a multivariable general linear model using MaAsLin2. Random effects included farm sample location and sampling time period.

We hypothesize that the environmental conditions of manure pits and time outside the anerobic gut result in a restructuring of the microbial communities and associated ARGs of fresh cow manure. We further hypothesize that the spraying of differentially microbiota-enriched manure from the manure pit may impact the microbial community composition of the agricultural soil. To evaluate these differences in microbial communities and antimicrobial resistance profiles across fresh manure, manure pits, and field soils, we collected manure and soil samples from 15 different dairy farms across Wisconsin (United States) in the fall of 2015 and 2016 and in the spring of 2016. We collected fresh manure, samples from 3 different depths of manure pits (6, 12, and 24 in.) and from 2 depths of field soil (6 and 12 in.) (Fig. 1A). To assess microbial community and functional ARGs, we used a combination of shotgun metagenomic sequencing and functional metagenomics to evaluate microbial taxonomic composition as well as known and novel ARG composition. Our unique approach of shotgun sequencing paired with functional metagenomics allowed us not only to address spread and transfer of antimicrobial resistance to environmental microbes at greater resolution than previously studied but also to survey potential novel resistance genes that may be arising in an understudied agricultural environment (2). We found that during the transitions from fresh manure to manure pits to field soil, the microbiome is characterized by distinct configurations—defined as differences in prevalence and abundance—in both taxonomic and ARG composition, with little evidence of manure ARGs transferring to field soil samples.

## RESULTS

### Taxonomic composition experiences a reconfiguration from fresh manure to manure pit samples, while soil samples are taxonomically distinct.

To compare taxonomic diversity between microbial communities, we performed shotgun metagenomic sequencing on all sample types and determined taxonomic abundances from processed reads using Kraken2 (39). Fresh manure had significantly higher alpha diversity, measured as richness (Fig. 1B) and Shannon’s H (see Fig. S1 in the supplemental material), compared to manure pits samples collected at various depths (6, 12, and 24 in.) (linear mixed-effects model [LME], *P* < 0.001). This difference in taxonomic diversity suggests a change in microbial communities after the transition from fresh manure to manure pits. When we compared diversity between soil and manure samples, we found that soil samples had a higher species richness and Shannon’s H than fresh manure or manure pit samples (Fig. 1B and Fig. S1) (LME, *P* < 0.05). We found no significant differences in either species richness or Shannon’s H when comparing manure at different depths or soil at different depths (Fig. 1B and Fig. S1).

10.1128/mBio.00798-21.1 FIG S1 Alpha diversity varied across fresh manure, manure pit, and soil samples. Box plot of alpha diversity using Shannon’s H by sample type. Points indicate each individual sample measured. Significance determined by linear mixed-effects model with random effects as location of sampling (marginal *R*^2^ = 0.478, conditional *R*^2^ = 0.621), followed by least-square means pairwise comparisons. Fresh manure was significantly different from manure pit samples (*P* < 0.001). Soil samples were significantly different from manure samples (*P* < 0.001). Download FIG S1, PDF file, 0.4 MB. Copyright © 2021 Sukhum et al. 2021 Sukhum et al. https://creativecommons.org/licenses/by/4.0/ This content is distributed under the terms of the Creative Commons Attribution 4.0 International license.

To compare microbial beta diversity between sample types, we calculated a Bray-Curtis dissimilarity index from species abundances and visualized dissimilarity indices using principal coordinate analysis (PCoA). We found significant differences in taxonomic composition based on both farm and season of collection (PERMANOVA: *P* < 0.05) (Fig. S2). We found that soil samples, regardless of depth, have a taxonomic composition that is significantly distinct from those of both fresh manure and manure pit samples after controlling for repeated measures of sampling location (permutational multivariate analysis of variance [PERMANOVA], *P* < 0.001) (Fig. 1C). Further, the taxonomic composition of fresh manure samples is significantly different from that of manure pit samples after controlling for repeated measures of sampling location (PERMANOVA, *P* < 0.001), while manure pit samples have wide compositional variation (Fig. 1C). When comparing pairwise Bray-Curtis dissimilarities within each sample types, we found that manure pit samples have significantly higher beta diversity than both fresh manure and soil samples (Fig. 1D) (*P* < 0.05), while soil samples had lower beta diversity than manure samples (Fig. 1D) (*P* < 0.001).

10.1128/mBio.00798-21.2 FIG S2 Taxonomic composition varied across farm and collection period. Principal-coordinate analysis (PCoA) plot of Bray-Curtis dissimilarity index for species abundances of all sample types. There is significant clustering by farm and collection period after controlling for repeated measures of sampling location (PERMANOVA, *P* < 0.001). Download FIG S2, PDF file, 0.1 MB. Copyright © 2021 Sukhum et al. 2021 Sukhum et al. https://creativecommons.org/licenses/by/4.0/ This content is distributed under the terms of the Creative Commons Attribution 4.0 International license.

Phylum abundances varied between soil and manure samples, with soil samples primarily being composed of *Proteobacteria* and *Actinobacteria*, while manure samples had high abundances of *Proteobacteria*, *Bacteroidetes*, and *Firmicutes* (Fig. S3). To evaluate significant taxonomic differences between soil and manure samples, we used multivariable association analyses that rely on general linear models in MaAsLin2 in R (40). We found 10 phyla that had greater abundance in soil than manure, including *Acidobacteria*, *Actinobacteria*, and *Proteobacteria* (Fig. 1E) (general linear model [GLM], *q* value < 0.05). We also found that five anaerobic genera, including *Bifidobacterium*, *Clostridium*, *Fusobacterium*, *Prevotella*, and *Bacteroides*, had higher abundances in manure samples than soil samples (Table S1) (GLM, *q* value < 0.05). Taxonomic composition between fresh manure and manure pit samples had more phyla that were not significantly different than between manure and soil samples (Fig. 1C; Fig. S3) (GLM, *q* value < 0.05). We found that fresh manure samples are associated with an enrichment of eight phyla, including *Firmicutes*; while manure pit samples are associated with an enrichment of nine different phyla, including *Proteobacteria* (Fig. 1F; Table S1) (GLM, *q* value < 0.05). Fresh manure samples are also associated with a higher abundance of *Enterobacteriaceae*, Staphylococcus aureus, Enterococcus faecium, and Clostridioides difficile, taxa that have human pathobionts and antimicrobial resistance determinants (Table S1) (GLM, *q* value < 0.05).

10.1128/mBio.00798-21.3 FIG S3 Phylum composition varied across sample type. Stacked bar plots for phylum abundances of samples organized by sample types. Download FIG S3, PDF file, 0.2 MB. Copyright © 2021 Sukhum et al. 2021 Sukhum et al. https://creativecommons.org/licenses/by/4.0/ This content is distributed under the terms of the Creative Commons Attribution 4.0 International license.

10.1128/mBio.00798-21.9 TABLE S1 Significant results from MaAsLin2 taxonomic and ARG models. Download Table S1, XLSX file, 0.2 MB. Copyright © 2021 Sukhum et al. 2021 Sukhum et al. https://creativecommons.org/licenses/by/4.0/ This content is distributed under the terms of the Creative Commons Attribution 4.0 International license.

Thus, alpha diversity, beta diversity, and taxonomic composition-based analyses indicate a clear shift in the microbial community composition from fresh manure samples to manure pit samples but no significant shifts in taxonomic diversity across manure pit depths. Soil samples had high alpha diversity and were compositionally similar to other soil samples across farms and seasons but were compositionally distinct from all manure samples.

### Antimicrobial resistance gene abundance and diversity were higher in manure samples than in soil samples.

**FIG 2:**
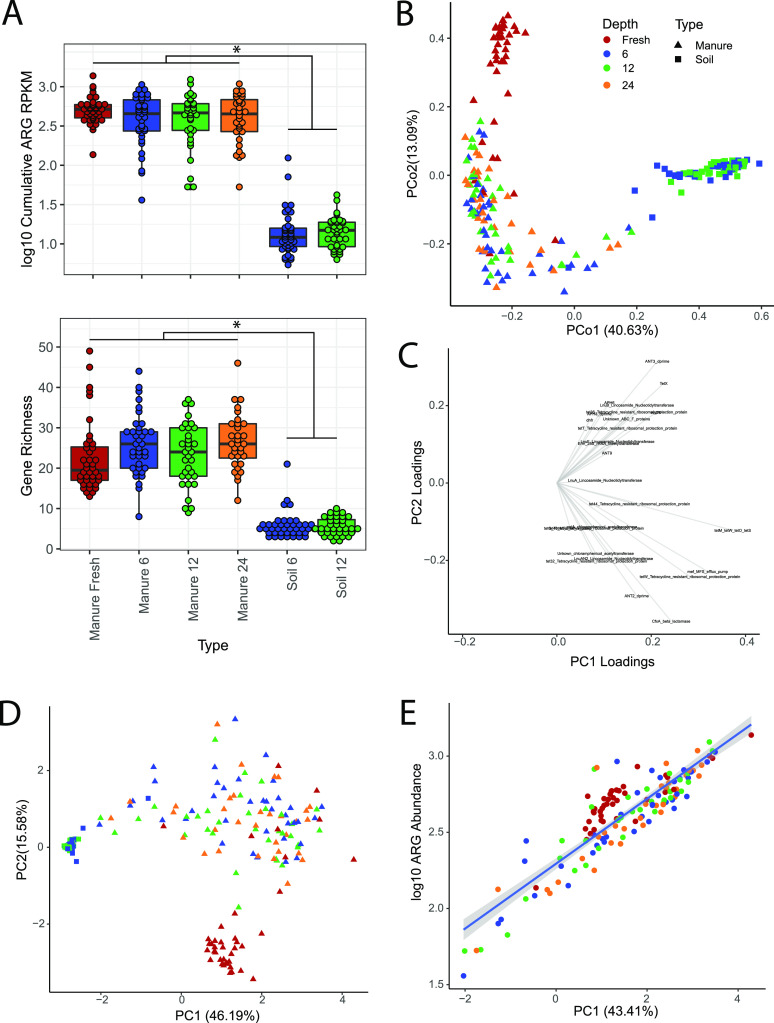
Antimicrobial resistance abundance and diversity varied across fresh manure, manure pit, and soil samples. (A) Box plots of ARG abundance and gene richness across sample type show a significant difference between manure and soil samples. Points indicate individual samples. Significance was determined by a linear mixed-effects model with random effects as location of sampling (for abundance marginal *R*^2^ = 0.658 and conditional *R*^2^ = 0.727; for gene richness, marginal *R*^2^ = 0.526 and conditional *R*^2^ = 0.690), followed by least-square means pairwise comparisons. Soil was significantly different from manure samples (*P* < 0.001). (B) Principal-coordinate analysis (PCoA) plot of the Bray-Curtis distance matrix for ARG abundances of all sample types. There is significant clustering by sample type after controlling for repeated measures of sampling location (PERMANOVA, *R*^2^ = 0.294, *P* < 0.001). (C) Principal-component analyses (PCA) eigenvectors with loading values with a <0.1 threshold for combined PCA of ARG and species abundances. (D) Scatterplot of PC1 and PC2 from PCA analysis of ARG and species abundances. (E) Scatterplot of PC1 against ARG abundance with a positive correlation by linear mixed-effects model with sample location as a random effect (estimate = 0.21, intercept = 2.30, marginal *R*^2^ = 0.781, conditional *R*^2^ = 0.866, *P* < 0.001).

While there are distinct taxonomic differences between fresh manure, manure pits, and soil, these communities do not tell us about shifts in antimicrobial resistance that may be associated with the dairy cow gut and potentially transferred to manure pits and soils. To determine functional antimicrobial resistance profiles, we created functional metagenomic libraries from 143 dairy manure metagenomes, representative of all manure pit depths and fresh manure. From those samples, we constructed nine functional metagenomic libraries, screened the libraries on 15 antimicrobials commonly used in agricultural animals, and recovered 130 selections of resistant transformants. Resistance screens on d-cycloserine, tetracycline, and trimethoprim yielded the highest colony counts (3,000) in all nine libraries (Fig. S4). Resistance-conforming inserts were amplified, sequenced, and assembled with the Parallel Annotation and Reassembly of Functional Metagenomic Selections (PARFuMS) pipeline (41). Antimicrobial resistance gene abundances (assessed as reads per kilobase of transcript per million mapped reads [RPKM]) were quantified in all processed reads using ShortBRED v0.9.4 (42) with a curated database of all resistance proteins from the Comprehensive Antibiotic Resistance Database (CARD) (43) combined with the functionally selected resistance proteins characterized in this study (Table S2) (41, 42). We used a rarefaction analysis to determine saturation of ARG richness based on read coverage for both manure and soil samples (Fig. S5). We found that manure samples contained a higher relative abundance (RPKM) and a greater diversity of ARGs than soil samples (Fig. 2A) (LME, *P* < 0.05). This difference may be reflective of differences in coverage between manure samples and soil samples (44). However, there were no significant differences in ARG abundance or diversity between fresh manure and the manure pit samples at any depth (Fig. 2A).

10.1128/mBio.00798-21.4 FIG S4 Beta diversity of resistance gene composition across sample types. Boxplot of resistome beta diversity determined by Bray-Curtis dissimilarity comparisons for each sample time. Points indicate pairwise comparison by sample type. Download FIG S4, PDF file, 0 MB. Copyright © 2021 Sukhum et al. 2021 Sukhum et al. https://creativecommons.org/licenses/by/4.0/ This content is distributed under the terms of the Creative Commons Attribution 4.0 International license.

10.1128/mBio.00798-21.5 FIG S5 Rarefaction analysis of resistance gene richness for manure and soil samples. Box plot of ARG richness by read depth at 500,000-read intervals. Download FIG S5, PDF file, 0.1 MB. Copyright © 2021 Sukhum et al. 2021 Sukhum et al. https://creativecommons.org/licenses/by/4.0/ This content is distributed under the terms of the Creative Commons Attribution 4.0 International license.

10.1128/mBio.00798-21.10 TABLE S2 Metadata and ShortBRED results from functional metagenomics output and exclusion criteria for resistome profiling. Download Table S2, XLSX file, 1.7 MB. Copyright © 2021 Sukhum et al. 2021 Sukhum et al. https://creativecommons.org/licenses/by/4.0/ This content is distributed under the terms of the Creative Commons Attribution 4.0 International license.

We compared ARG composition between fresh manure, manure pits, and soil samples using Bray-Curtis dissimilarity from ARG abundances and PCoA. We found that, similar to the taxonomic PCoA, soil samples cluster separately from both fresh manure and manure pit samples (PERMANOVA, *P* < 0.001) (Fig. 2B). When we compared beta diversity for resistance composition, we found that pairwise Bray-Curtis dissimilarities were higher in soil samples than in manure samples and that fresh manure had significantly lower resistome beta-diversity than any manure pit samples (Fig. S6) (LME, *P* < 0.001). Thus, while there is not a decrease in ARG abundance or diversity from fresh manure to manure pits, there is a reconfiguration of ARG composition after this transition. However, soil samples remain distinctly different from manure samples in both abundance and composition of ARGs.

10.1128/mBio.00798-21.6 FIG S6 PC1 is correlated with species richness across samples. Scatterplot of PC1 against species richness with a positive correlation by a linear mixed-effects model with sample location as random effect (estimate = 9.33, intercept = 105.14, marginal *R*^2^ = 0.140, conditional *R*^2^ = 0.651, *P* < 0.001). Download FIG S6, PDF file, 0.6 MB. Copyright © 2021 Sukhum et al. 2021 Sukhum et al. https://creativecommons.org/licenses/by/4.0/ This content is distributed under the terms of the Creative Commons Attribution 4.0 International license.

To integrate and compare both taxonomic and resistance composition differences between samples, we performed a Procrustes analyses on the Bray-Curtis distance matrices of species abundance (Fig. 1C) and ARG abundance (Fig. 2B). Procrustes analysis indicated that the microbial taxonomic and resistance structure are correlated (Procrustes sum of squares = 0.574; correlation in a symmetric Procrustes rotation = 0.653; *P* < 0.001). Subsequent principal-component analysis (PCA) on the combined matrices of resistance abundance and species abundance found that the variables with the highest loadings for both PC1 and PC2 were all ARGs, with 10 of the top 27 variables on PC1 corresponding to tetracycline resistance elements (Fig. 2C). Plotting PC1 and PC2 illustrates a structure similar to that seen with both taxonomic and resistance PCoA plots, where soil clusters tightly and separately from manure samples along PC1 and fresh manure clusters separately from manure pit and soil samples (Fig. 2D). Further, when comparing PC1 to ARG abundance or species abundance, we found a strong correlation between ARG abundance and PC1 (Fig. 2E) (GLM, *R*^2^ = 0.781, *P* < 0.001), and a weaker correlation between species abundance and PC1 (Fig. S7) (GLM, *R*^2^ = 0.140, *P* < 0.001). This suggests that the variation we found across this data set is highly correlated with total ARG abundance.

10.1128/mBio.00798-21.7 FIG S7 ARG counts across sample types by drug class. Total ARG counts as log(RPKM) of 17 identified drug classes for each sample type. Download FIG S7, PDF file, 0.1 MB. Copyright © 2021 Sukhum et al. 2021 Sukhum et al. https://creativecommons.org/licenses/by/4.0/ This content is distributed under the terms of the Creative Commons Attribution 4.0 International license.

Thus, we find that as the manure microbiome is transferred from fresh manure to manure pits, there is a change in the composition but not the abundance of ARGs. Furthermore, significant compositional differences in ARGs between manure and soil samples show little support for a shared resistance community or antimicrobial burden between sample types.

### Tetracycline, aminoglycoside, and MLS resistance determinants were enriched in manure samples compared to soil samples.

**FIG 3:**
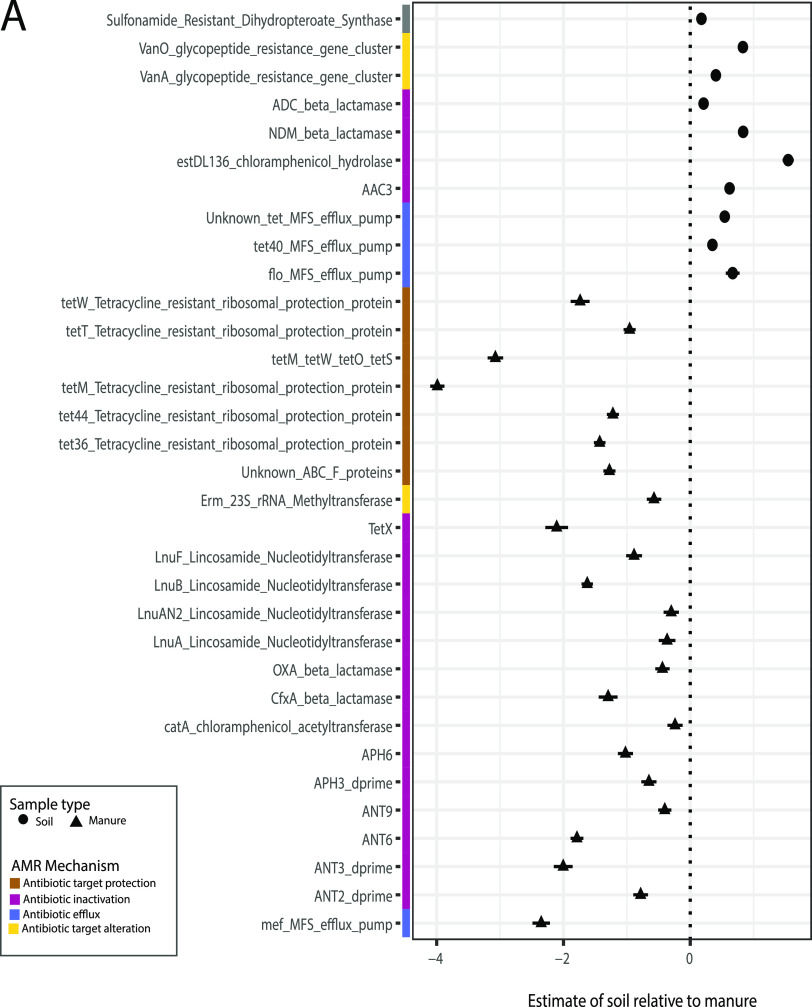

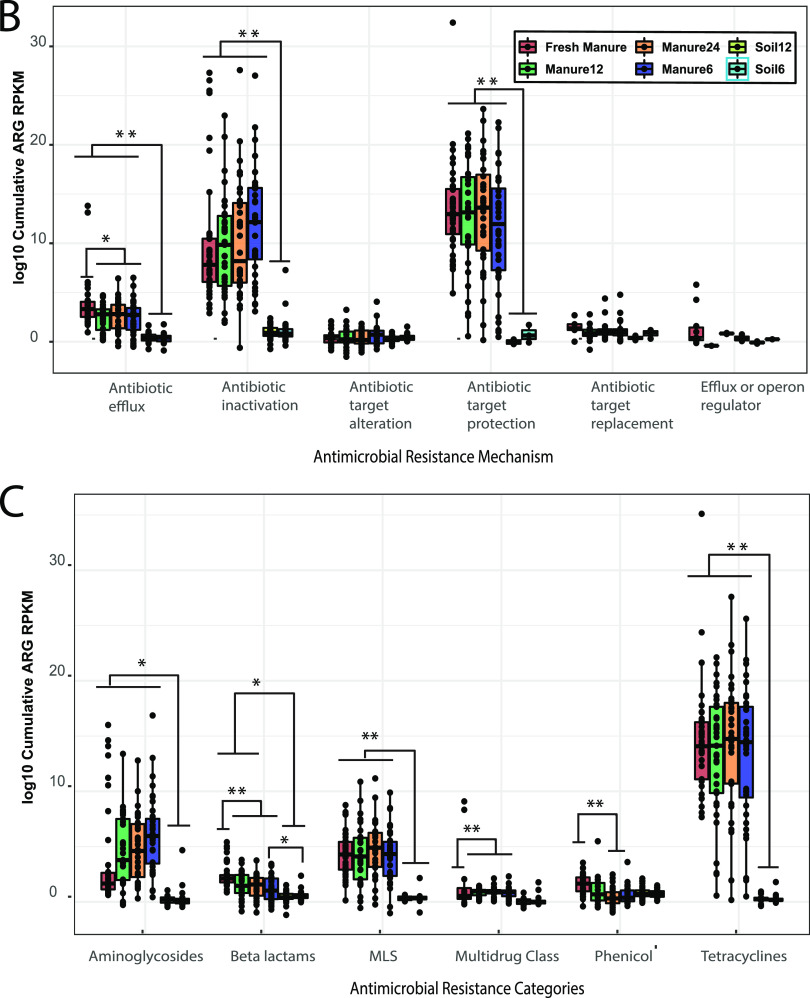
Resistance genes differ significantly between soil and manure samples. (A) Estimates of coefficients of soil samples relative to manure samples for significant resistance gene differences in a multivariable general linear model using MaAsLin2. Random effects included farm sample location and sampling time period. (B) Box plot of total resistance gene count [log_10_(RPKM)] grouped by antimicrobial resistance mechanism. Points indicate each individual sample measured. (C) Box plot of total resistance gene count grouped by the top six antimicrobial resistance categories. Significance was determined by a linear mixed-effects model with random effects as location of sampling (ARG resistance categories, marginal *R*^2^ = 0.68, conditional *R*^2^ = 0.749; antimicrobial resistance categories, marginal *R*^2^ = 0.670612, conditional *R*^2^ = 0.723), followed by least-square means pairwise comparisons. *, *P* < 0.03; **, *P* < 0.005.

To evaluate significant ARG class and family differences between soil and manure samples, we used multivariable association analyses using MaAsLin2 (40). Tetracycline, aminoglycoside, and macrolide-lincosamide-streptogramin (MLS) resistance genes had significantly different ARG abundances between manure (both fresh and pit) and soil samples (Fig. 3; Fig. S8) (LME, *P* < 0.05). Manure samples were significantly enriched in ARGs with antimicrobial inactivation, target protection, and efflux mechanisms (Fig. 3B) (LME, *P* < 0.01). Beta-lactam resistance determinants were significantly enriched in manure samples compared to soil samples at most depths (Fig. 3C) (LME, *P* < 0.05). Multidrug class resistance determinants were significantly different between fresh manure samples and soil samples at any depth (Fig. 3C) (LME, *P* < 0.01). ARGs that were enriched in soil samples compared to manure samples include vancomycin, *ADC* beta lactamase, *NDM* beta lactamase, *estDL136*, and *AAC3* genes (Fig. 3A; Table S1) (GLM, *q* value < 0.05). ARG mechanisms that were not significantly different between fresh manure, manure from manure pits, and field soil were antimicrobial target replacement, target alteration, and efflux/operon regulator mechanisms. Further, soil sample depth was not a significant factor in either ARG mechanism or drug target type, while manure sample depth was significant with phenicol ARGs (Fig. 3B and C). We observed that soil samples differ in their resistance profiles compared to all manure samples in their drug target ARG abundances. Resistance determinants and mechanisms varied between soil and manure regardless of depth; however, significant differences were observed between the resistomes of fresh manure and manure pits.

10.1128/mBio.00798-21.8 FIG S8 Colony counts of antimicrobial resistance screens. A heat map of log(colony counts) for 130 functional screens of 9 libraries across 15 antimicrobials. Download FIG S8, PDF file, 0.1 MB. Copyright © 2021 Sukhum et al. 2021 Sukhum et al. https://creativecommons.org/licenses/by/4.0/ This content is distributed under the terms of the Creative Commons Attribution 4.0 International license.

### Antimicrobial resistance profiles varied between fresh manure and manure pit samples.

Fresh manure and manure pit resistance profiles all contained high ARG abundances for aminoglycosides, beta-lactams, MLS, and tetracycline resistance and had similar ARG mechanism profiles (Fig. 3B and C). However, we found that beta-lactam ARG abundances were significantly different between fresh manure and manure pits at all depths (Fig. 3C) (LME, *P* < 0.05). Phenicol resistance gene abundance was significantly different between fresh manure and manure pit at depths of 6 and 24 in. (Fig. 3C) (LME, *P* < 0.01). Fresh manure had statistically significantly higher ARGs with antimicrobial efflux mechanisms than manure pits at different depths (Fig. 3B) (LME, *P* < 0.05). ARG mechanism profiles between fresh manure and manure pits were varied, and differences between sample types were also apparent at the gene product level.

**FIG 4:**
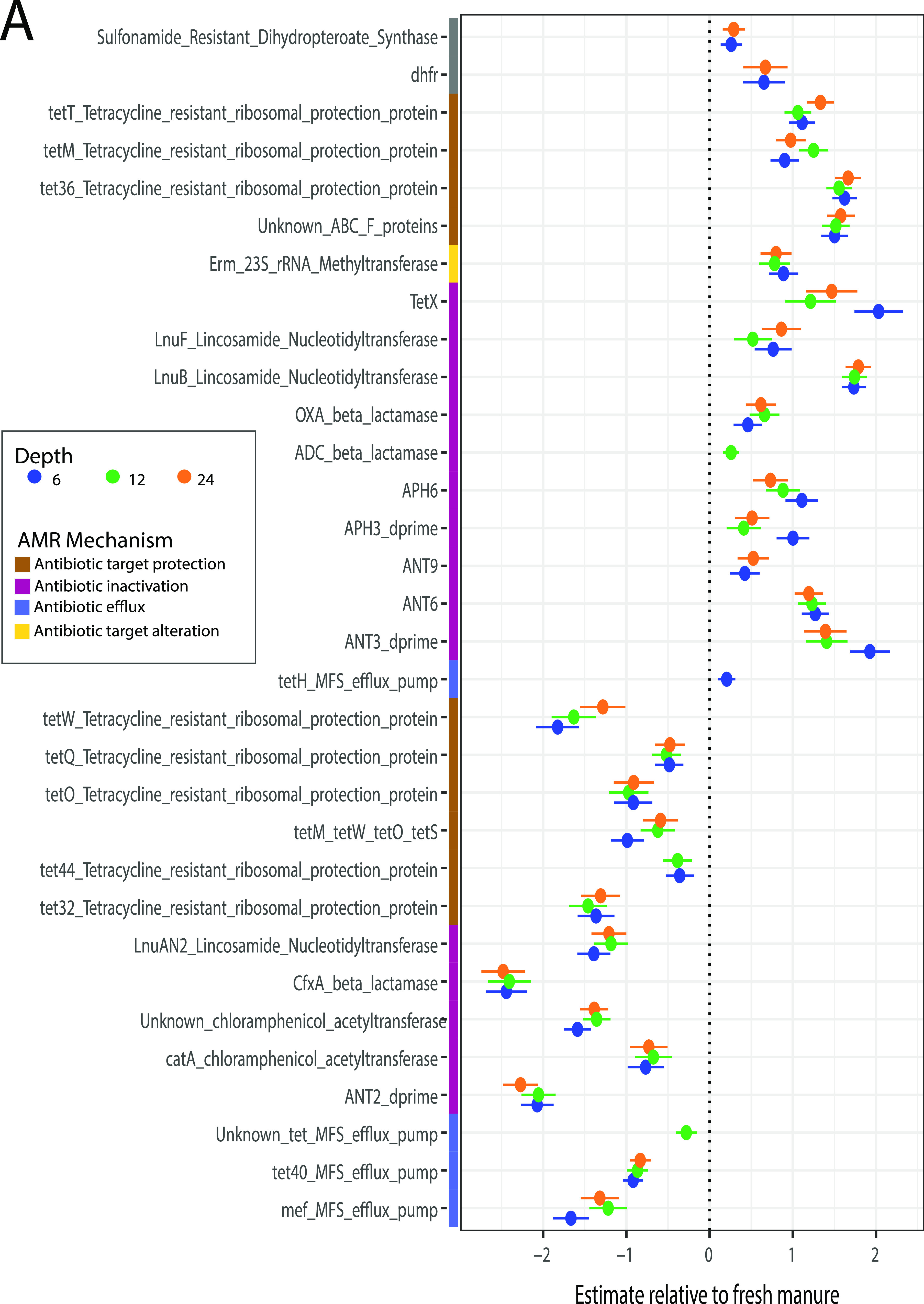

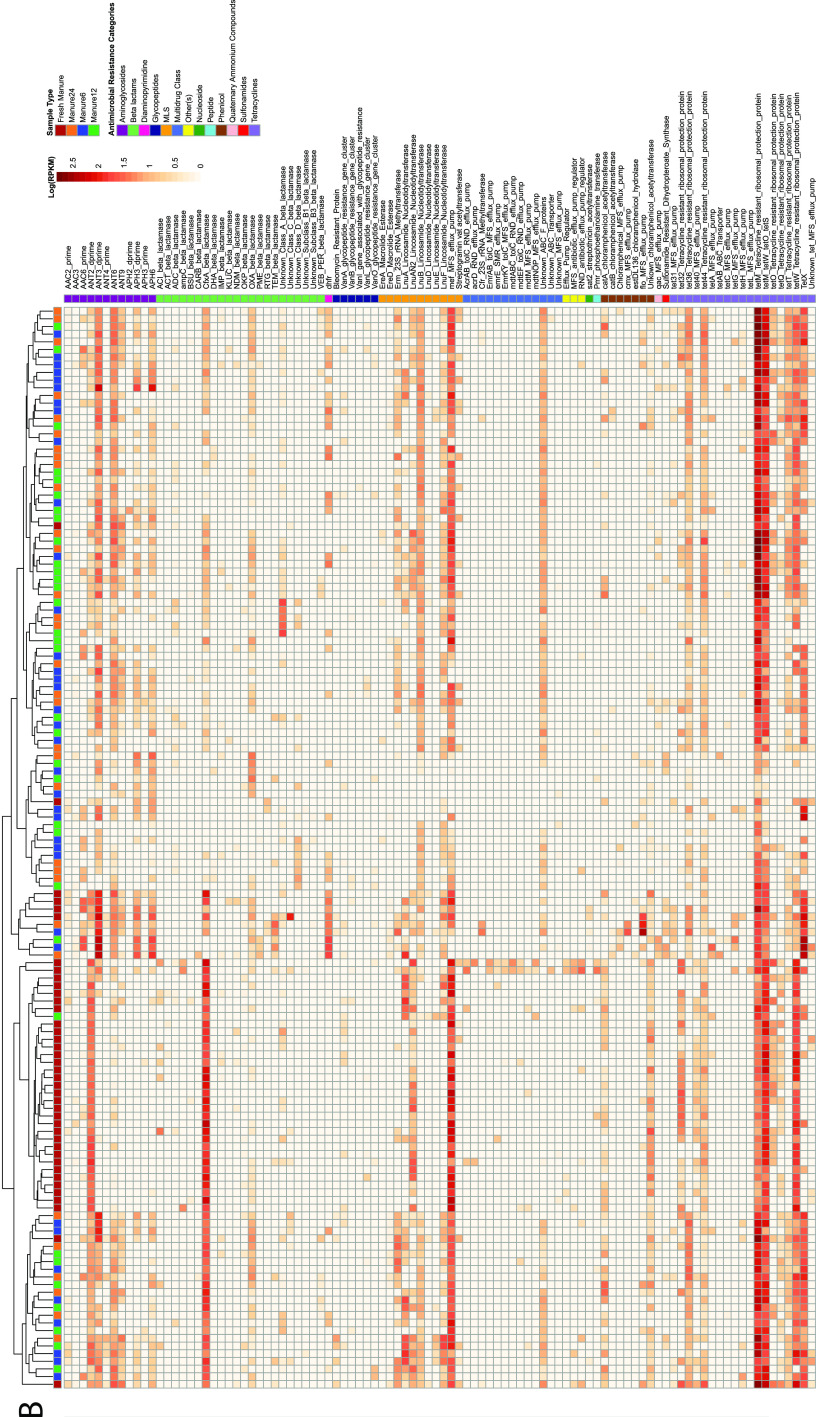
Resistance genes differ between fresh manure and manure pit samples. (A) Estimates of coefficients of manure pit depths relative to fresh manure for significant resistance gene differences in a multivariable general linear model using MaAsLin2. Random effects included farm sample location and sampling time period. (B) Resistance genes are grouped by antimicrobial class on the *y* axis and manure samples are hierarchically clustered by sample type on the *x* axis. Colored annotations indicate gene antimicrobial resistance category. Resistance gene count is presented as log_10_(RPKM). Hierarchical clustering was created in R using the package pheatmap (75).

To further characterize the differences between manure pit depths relative to fresh manure, we conducted multivariable association analyses. Manure pit profiles contained a wider range of enriched genes, such as *inuB*, *tetT*, *tetW*, and *tetX*, and were enriched in *tetM* products compared to fresh manure (Fig. 4A; Table S1). Fresh manure contained higher counts of *tetW*, *tetH*, *tetQ*, *tet40*, *cfx*, *mef*, *ANT2*, and *catA* genes than manure pits (Fig. 4A; Table S1). A wider variety of ARGs that mapped to glycopeptide, multidrug class, aminoglycoside, and tetracycline drug targets were characterized in manure pit samples than fresh manure (Fig. 4B). Manure pit samples were significantly enriched with ARGs with antimicrobial inactivation and antimicrobial target alteration mechanisms relative to fresh manure, while fresh manure had significantly more ARGs with antimicrobial efflux and target protection mechanisms (Fig. 4A; Table S1) (GLM, *q* value < 0.05). ARGs varied between fresh manure and pit manure in both ARG drug targets and mechanisms.

## DISCUSSION

The mammalian gut is a known reservoir of pathobionts and antibiotic resistance elements (45). Dairy farms routinely fertilize their fields using cow manure, after a period of storage in manure pits. The application of manure from cow manure pits to field soil may facilitate spread of the dairy cow gut microbial community and its associated ARGs to the broader environment (14, 46). To determine the extent of both taxonomic and resistome similarity during these transitions, we sampled fresh manure, manure pits, and field soil across 15 different farms. We found that as microbial communities transition from fresh manure to manure pits, the taxonomic composition and resistance profiles experience distinct reconfigurations but not an overall decrease in resistance gene abundance; furthermore, there was relatively low taxonomic and resistome similarity between manure and field soil, suggesting that application of pit manure does not contribute substantially to the soil microbiome or resistome at the interrogated resolution.

When comparing microbial communities between manure pits and field soil, we found large taxonomic composition differences that suggest that the field soil microbiome is resilient to microbial perturbation from the manure microbiome and that soils remained distinct from manure samples and similar to control soil samples, despite having manure from the manure pits introduced regularly. In further support of this dominant resiliency of the soil microbiome, despite the fact that field samples were collected across seasons and a variety of farms and field types, soil samples were compositionally more similar to each other than to manure pit samples. As manure pit samples showed high beta-diversity between samples, we would have also expected beta diversity between field soils to increase after manure was spread over field soils if there were significant transfers of manure microbes to the soil; however, low beta diversity of soil samples suggests that little perturbation of field microbial communities resulted from manure spreading. Additionally, as manure is spread across the top of the soil, we might expect to find variation in the microbial communities at different depths, with manure more strongly impacting the top layer of soil than the lower level (47). Instead, soil communities were similar in all taxonomic community measurements regardless of depth, suggesting a limited impact of manure spreading on taxonomic composition, even close to the surface. Our results, paired with results in recent literature, suggest the need for studies at the level of microbial strains and functions to assess whether manure application affects soil microbial communities and ARGs found in the environment at deeper resolution and lower frequencies than we and others have so far performed, in both short and long time periods (23, 48, 49).

When microbial communities transition between fresh manure to manure pits, the communities experience taxonomic shifts that are likely part of a fundamental restructure that occurs as the community transitions from a gut to an environmental structure. During this transition, we would expect at least three possibilities: taxa that are gut specific to be outcompeted, taxa that are able to survive environmental conditions to be selected, and new taxa from the environment to be introduced. The shifts we found in taxa support these possibilities. We found a decrease in *Enterobacteriaceae*, a family known for its diverse pathobionts that are often found in the gut, in pathobiont species such as S. aureus, E. faecium, and C. difficile, and in anaerobic genera such as *Actinomyces*, *Fusobacterium*, *Clostridium*, *Bacteroides*, *Prevotella*, and *Bifidobacterium* (50). These changes may indicate a shift away from pathobionts and gut-associated bacteria. In the manure pits, we found an increase in *Firmicutes*, which can be found in various environments and can survive extreme conditions. We also found an increase in *Actinobacteria*, which include terrestrial and aquatic bacteria that are less present in gut communities and particularly enriched in our soil samples (51). Both phyla might suggest a shift to a more environmental microbial composition.

Although there is a taxonomic reconfiguration from fresh manure to manure pits with a decrease in species alpha diversity, we did not find a decrease in abundance or richness of ARGs. This is especially interesting because we might expect the selective pressures in the mammalian gut that enrich ARGs to be decreased in environmental settings. Lack of ARG depletion suggests that either ARGs are maintained outside the gut, possibly by stable plasmids or by selective pressures from the excreted antimicrobials that are not metabolized, or ARGs are introduced to the manure pits from sources other than the dairy cow gut (18, 30, 32). Further, we found a reconfiguration in the composition of ARGs, with specific ARGs being enriched in either fresh manure or manure pits and an increase in beta diversity between fresh manure and manure pits. These compositional shifts may be a consequence of taxonomic shifts impacting which ARGs are prevalent in manure pits.

ARG profile might be related to particular antimicrobials that dairy cows are given when sick. We observed high counts of ARGs conferring resistance to tetracyclines, aminoglycosides, and beta-lactam in manure samples. Manure ARG counts were significantly different from those in soil, and ARGs varied between fresh manure and pit manure in both drug targets and mechanisms. Additionally, while most fresh manure samples clustered together, a few samples stand out distinctly as being more similar to manure pit samples than to fresh manure samples. As these fresh samples are compositionally distinct, their unique diversity and abundance could contribute to and drive the diversity we find in manure pits, which is inherently a slurry of many different fresh manure samples over time. We do not know the health or state of the cows that these fresh manure samples came from, but it is possible that some may have been clinically treated with antimicrobials, which might explain their unique ARG profiles. The particular enrichment in manure samples of tetracycline and aminoglycoside resistance determinants might occur because both drug classes are commonly used to treat mastitis and other susceptible bacterial infections in agricultural animals (13, 52, 53). However, increased resistance limits the efficiency of tetracycline and aminoglycosides in agricultural and clinical settings due to a variety of inactivating mechanisms (54–56). Further, there is a high risk of resistance genes with novel resistance activities emerging in response to antimicrobial pressures. These novel activities are commonly observed in broad-spectrum tetracycline resistance (54, 57, 58). *tet*(X) was detected in high abundances in manure pit samples. Monitoring the prevalence of *tet*(X), especially in the environment, is a priority considering its expanded substrate range, which poses a threat to human and animal health (59). To accurately characterize changes in ARGs with consistent manure applications, future studies should focus on longitudinal sampling (60). Further, recent research suggests that soil type, pH, and nutrient availability influence microbial compositional changes and ARGs in the environment (61, 62). Future studies could incorporate this information to better understand compositional shifts.

In conclusion, our approach of investigating longitudinal and comparative microbiome and ARG changes in manure over transitions from fresh manure to short- and long-term storage in manure pits to its application as fertilizer in agricultural fields provides a comprehensive microbiome and ARG characterization to the field of animal and environmental microbiome studies, with its potential implications for human health. Further, our unique approach of shotgun metagenomic sequencing combined with functional metagenomics provides a comprehensive and detailed description of both taxonomic and resistome variation based on a longitudinal study of 15 small, medium, and large dairy farms in Wisconsin, a major dairy state of the United States. We found that as the manure microbiome transitions from fresh dairy cow manure to manure pits, microbial taxonomic compositions and resistance profiles experience distinct reconfigurations that potentially correspond to the fresh manure going from a gut-structured community to an environment-structured community. Further, when we compared manure to field soils, we did not find evidence of shared community or a transfer of ARG abundance, with the field soil microbiome remaining relatively unperturbed by manure spread. Thus, interest in decreasing the spread of pathogenic bacteria and ARGs in an agricultural setting might focus effort and further studies on disrupting microbial communities in fresh manure and manure pits to decrease ARG abundance. Some ways of doing this might include removing selective pressures for ARG maintenance, such as remnant antimicrobials, and incorporating proper manure storage buffers to prevent runoff. However, our study suggests that the risks of ARGs spreading to field soil from manure pits are limited, at least at the resolution and frequency sampled in our study.

## MATERIALS AND METHODS

### Sample collection.

Fresh manure, manure stored in manure pits, and soil samples were collected from the same set of 15 Midwestern dairy farms over three seasons, fall 2015, spring 2016, and fall 2016. For the fall 2015 collection, samples were collected over 4 days during 2 days in last week of August and second week of September. The spring 2016 samples were collected during the third week of April, and fall 2016 samples were collected during first and second weeks of September. Fresh manures samples were collected either catching during defecation or from the core of the freshly defecated manure pile next to a cow. Samples from the holding tanks were collected at depths of 6, 12, and 24 in. from the most convenient and safest edge in 50 ml conical flasks. Holding tanks are small (∼20 feet diameter) to large (∼50 feet) pond-like reservoirs where fresh manures are drained off throughout the year for collection and then used and spread in the fields (corn or soybean) at the end of fall harvest or at the beginning of the spring planting season. Two sets of soil samples at depths of 6 and 12 in. were collected from either corn or soybean fields where the manures had been spread once approximately 6 months prior. Both manure and soil samples were collected using aseptic collection techniques in sterile 50-ml conical tubes or 50-ml wide-mouth plastic cups with lids, transferred to a cooler filled with dry ice, transported to laboratory, and stored in a −80°C freezer until further processing. Farms included small (<24 cows), medium (>25 to 100 cows), and large (>100 cows) farms.

### DNA extraction and sequencing.

Total genomic DNA was extracted from ∼100 mg of each manure and soil sample using the DNeasy Powersoil kit (Qiagen, Germantown, MD, USA). The kit protocol was used with a minor modification, where samples were lysed using a Mini-Beadbeater 24 (Biospec Products) rather than a vortex adapter as described previously (63, 64). We quantified DNA concentration using Qubit fluorometer double-strand-DNA (dsDNA) assays (Thermo Fisher Scientific). Illumina sequencing libraries were prepared using 0.5 ng/μl of genomic DNA in a modified Nextera kit protocol (Illumina, San Diego, CA, USA), following the modifications described by Baym et al. (65). We pooled and sequenced libraries on a NextSeq HighOutput platform (Illumina) to obtain ∼5 million 2 × 150-bp reads per sample. Illumina paired-end reads were demultiplexed by barcode. Adapters were removed using Trimmomatic v36 (Illuminaclip = 2:30:10:1:TRUE, Leading = 10, Trailing = 10, Sliding Window = 4:15, Min Length = 60), and contaminating human and cow reads were removed using Deconseq v0.4.3 (66, 67).

### Taxonomic metagenomics and analyses.

Microbial taxonomic composition and abundances were determined from decontaminated shotgun reads using Kraken2 and relative abundances were calculated using Bracken (39, 68). Species richness, Shannon’s H, and Bray-Curtis distances were computed in R using vegan (69). PERMANOVA were calculated using the adonis2 function of the vegan package in R with population groupings indicated by strata (69). Statistical modeling was conducted using linear mixed-effects models to account for repeat measuring for farms using lme4 and MaAsLin2 in R, with sample type as fixed effect and farm ID and collection period as random effects (40, 70).

### Functional metagenomics and analyses.

Manure and soil libraries were screened for resistance against 15 antimicrobials (aztreonam, cefepime, cefotaxime, cefoxitin, ceftazidime, chloramphenicol, colistin, d-cycloserine, doxycycline, gentamicin, penicillin, piperacillin, tetracycline, tigecycline, and trimethoprim) representing eight drug classes (β-lactams, tetracyclines, aminoglycosides, amphenicols, quinolones, sulfonamides, polymyxins, and cycloserine) using previously described methods (71–73). Nine libraries of approximately 15 samples each were created by pooling 143 manure samples, and resistance was observed in all antimicrobials screened to make a total of 130 selections of resistant transformants for further use. Resistance-conferring fragments were sequenced and assembled into contigs using the Parallel Annotation and Reassembly of Functional Metagenomic Selections (PARFuMS) v1.1 assembly pipeline (41). Contigs exceeding the colony count of the library by 10× were filtered from downstream analyses. To obtain a comprehensive list of ARGs and identify the abundance of resistant genes, contig open reading frames (ORFs) were then screened using several databases (CARD, Resfinder, and NCBI_AMR) in a hierarchical fashion. Databases were combined for a total of 6,594 markers and 2,314 ARG gene families utilized. Nonredundant annotations from functional metagenomic screening with known curated databases (CARD and NCBI-AMR) were used for resistance identification. Marker identification was used with Uniref90 and clustered at 95% identity for marker creation, and resistance gene markers in our shotgun metagenomic samples were identified using ShortBRED v0.9.4 (42). We manually filtered marker file for false positives (Table S2 for exclusion criteria). Statistical analysis was performed on the resulting 409 ARG families using PERMANOVA and linear mixed-effects models using the Adonis and lme4 R packages as described above. Taxonomic and resistome PCoA were compared and visualized with the Procrustes function in the vegan R package (69).

### Resistance metagenomics and analyses.

Antimicrobial resistance genes were identified using ShortBRED v0.9.4 (42) from decontaminated shotgun reads as described above using ARGs from the CARD and NCBI-AMR database. Similar to taxonomic metagenomic analysis, ARG composition, abundance, gene richness, Shannon’s H, and Bray-Curtis distances were computed using the vegan R package (69). We used a rarefaction analysis to determine saturation of ARG richness based on read coverage for both manure and soil samples by subsampling reads from our original samples at intervals of 500,000 reads. PERMANOVA and linear mixed-effects models were calculated using the adonis2 function in vegan and lme4 in R, respectively (69, 70). Multivariable association analyses were conducted using MaAsLin2 in R (27). Sample type was set as a fixed effect and farm ID and collection period as random effects for the linear mixed-effects models and multivariable association analyses (40, 70). Procrustes analyses of taxonomy and ARG composition were performed with the Procrustes function in the vegan package in R with 10,000 permutations. To visualize differences between a combined taxonomic and ARG matrix, we used a principal-component analysis (PCA) using labdsv in R (74). We did not transform combined data using the Bray-Curtis dissimilarity as was done in PCoA previously.

### Data availability.

Shotgun metagenomic reads generated for this study were uploaded to NCBI under the BioProject no. PRJNA671703 (http://www.ncbi.nlm.nih.gov/bioproject/671703).
