# Supplementary figures and images for: Manure Microbial Communities and Resistance Profiles Reconfigure after Transition to Manure Pits and Differ from Those in Fertilized Field Soil
Source: mBio. 2021 May 11;12(3):e00798-21. doi: 10.1128/mBio.00798-21 (PMC8262906; doi:10.1128/mBio.00798-21)

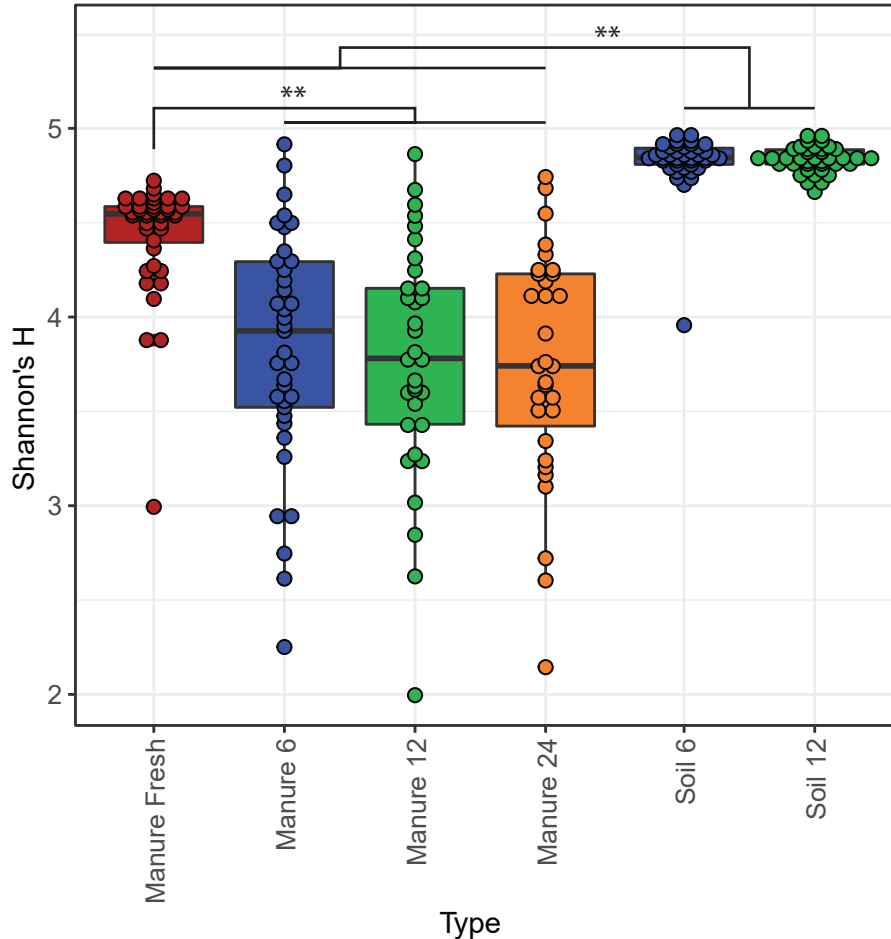

Supplement: FIG S1 [file mbio.00798-21-sf001.pdf]

A

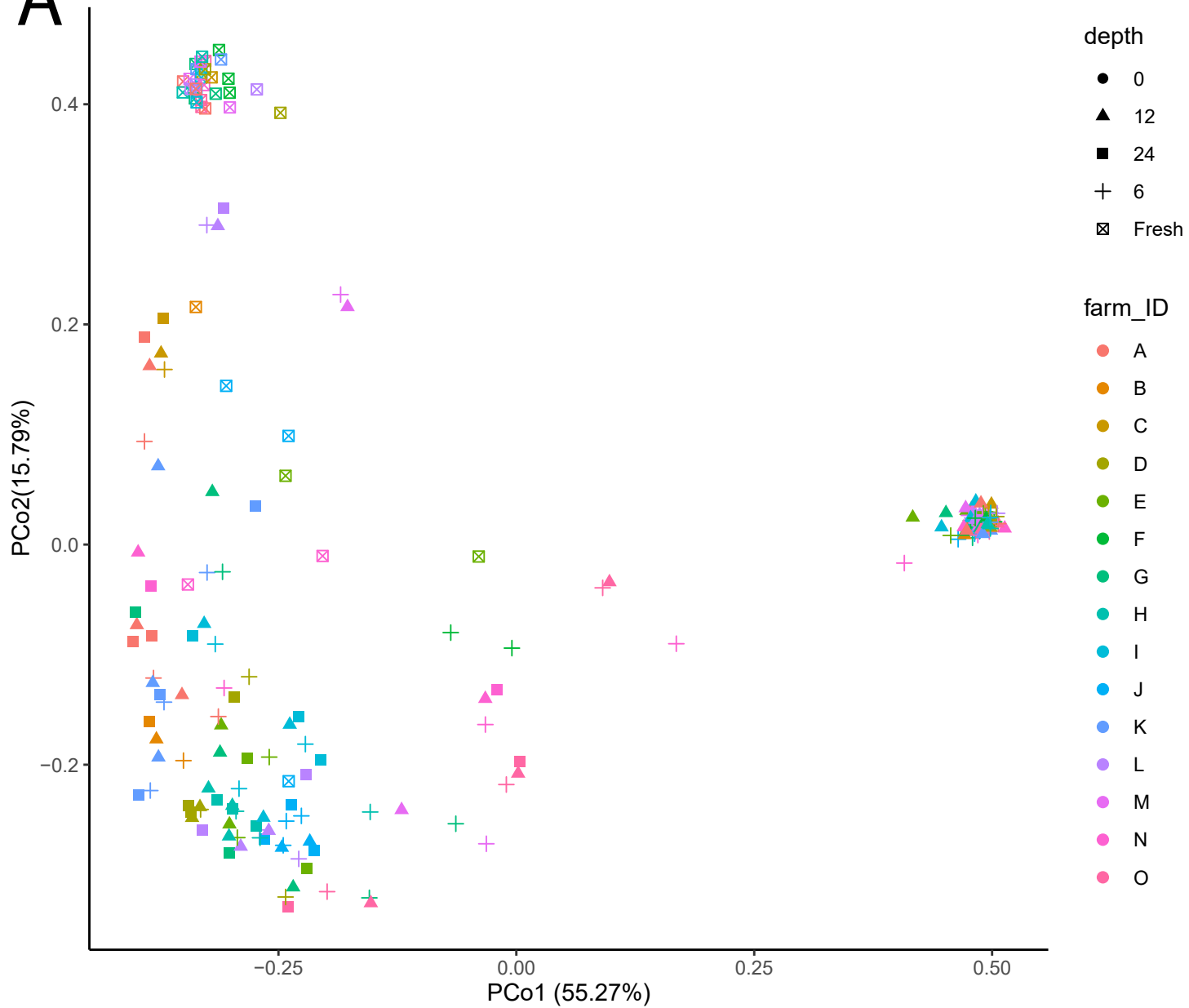

B

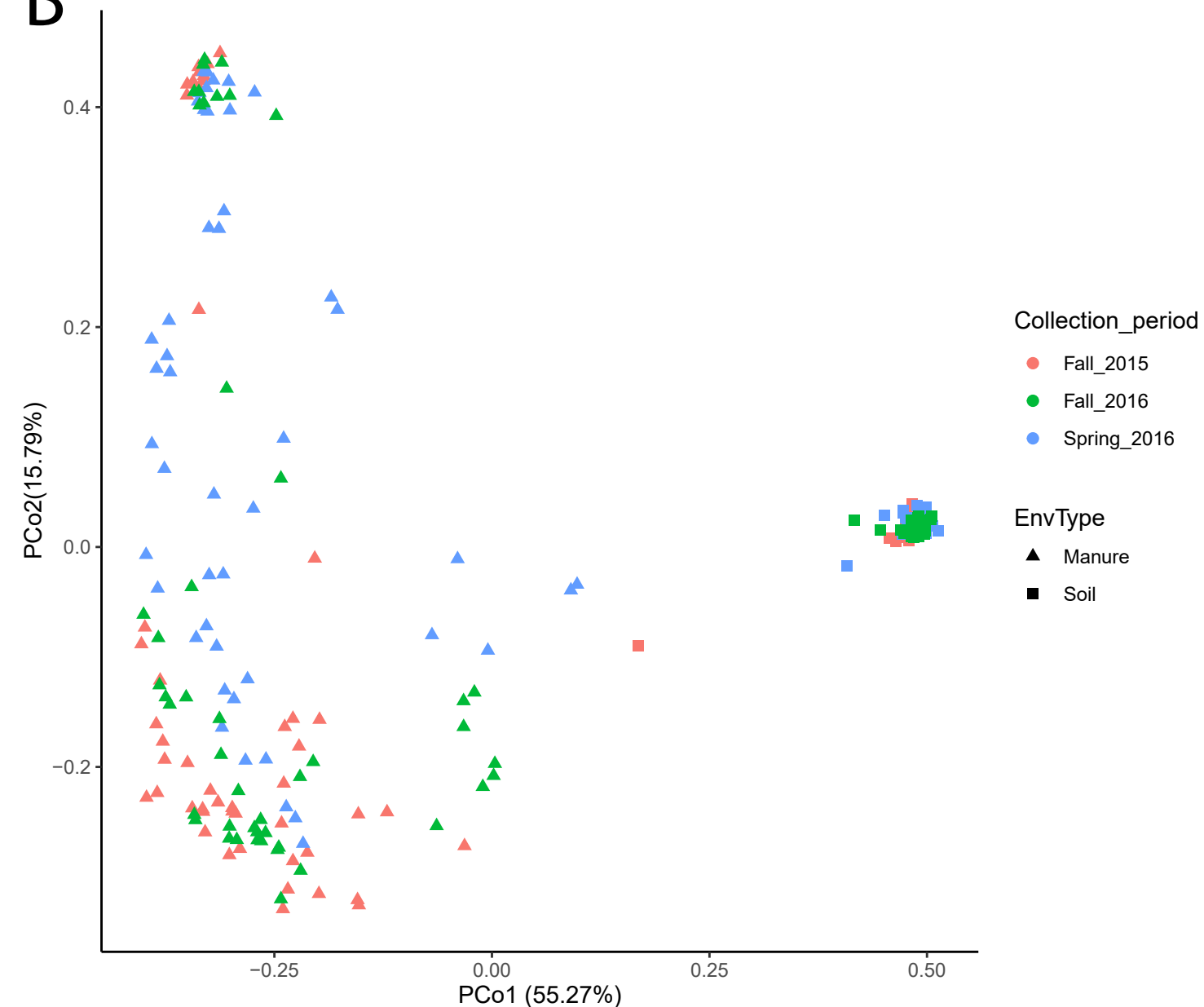

Supplement: FIG S2 [file mbio.00798-21-sf002.pdf]

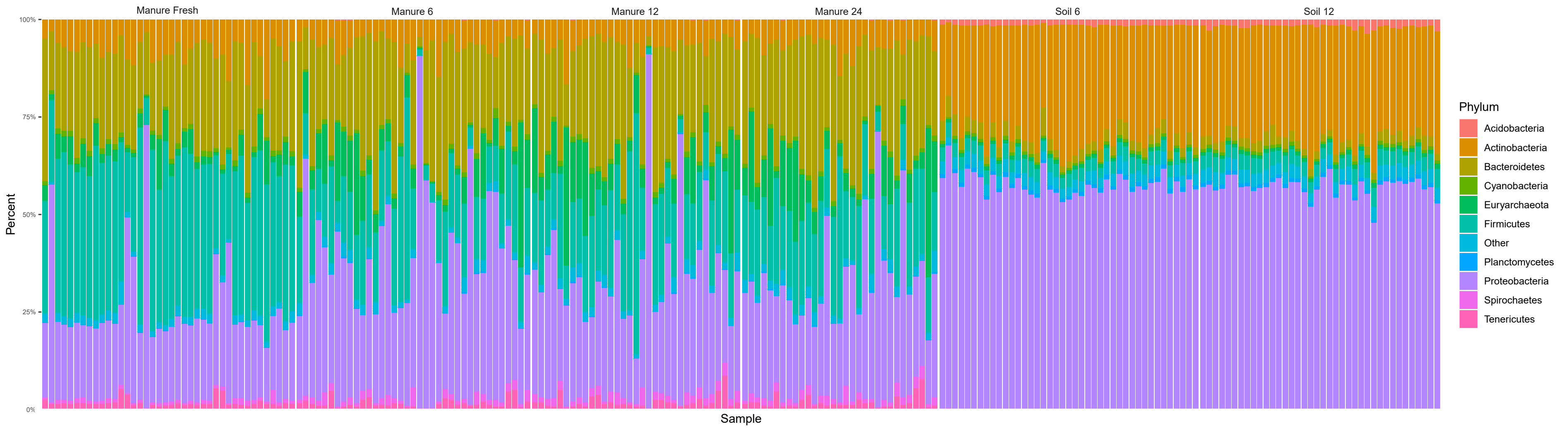

Supplement: FIG S3 [file mbio.00798-21-sf003.pdf]

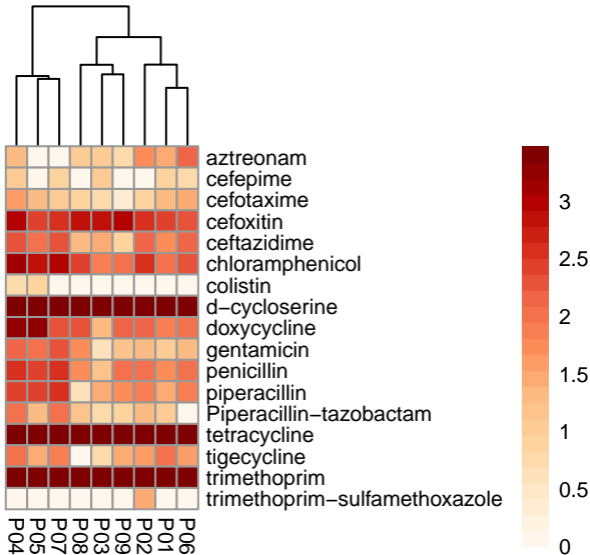

Supplement: FIG S4 [file mbio.00798-21-sf004.pdf]

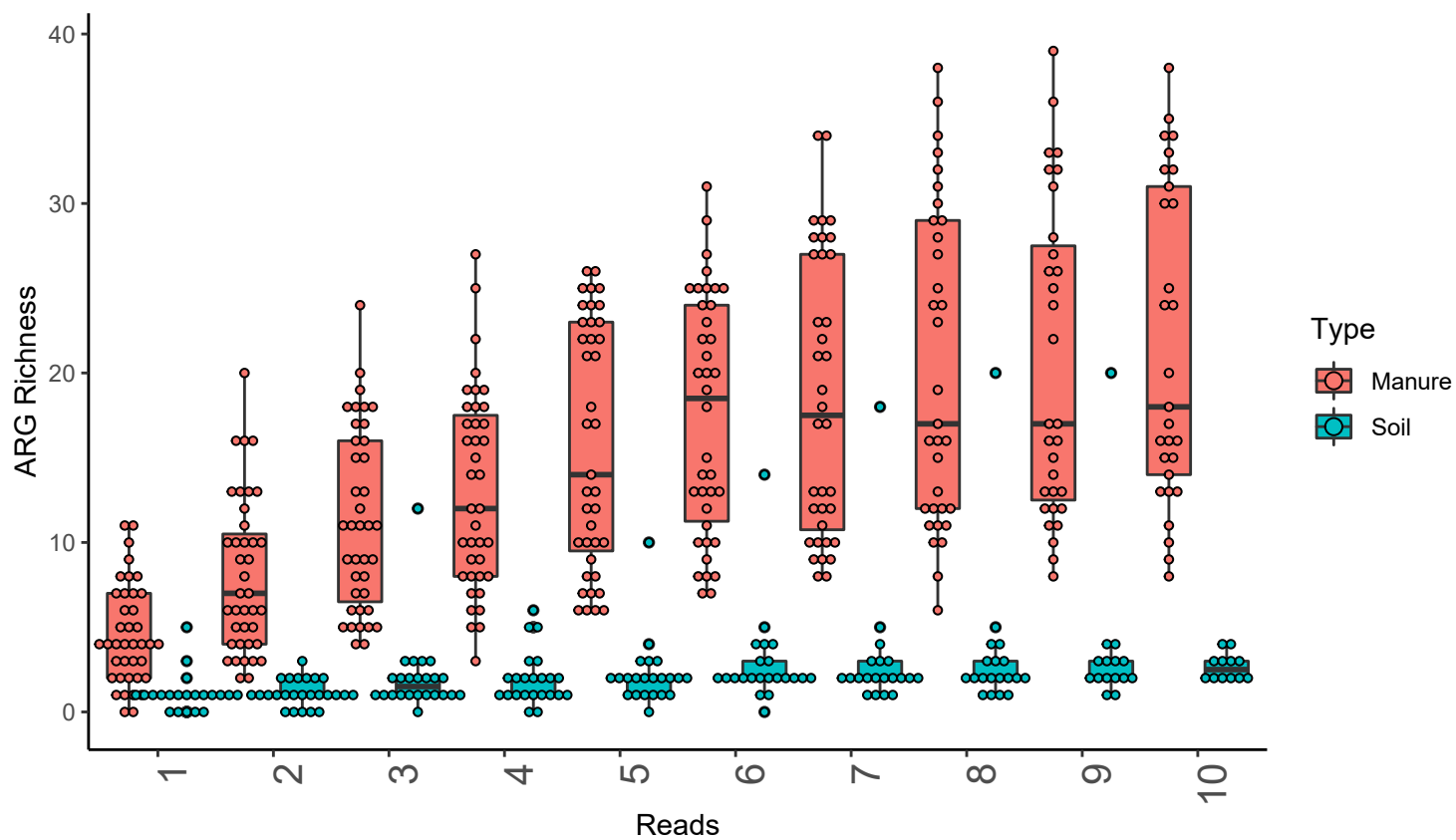

Supplement: FIG S5 [file mbio.00798-21-sf005.pdf]

Bray Curtis Beta Diversity

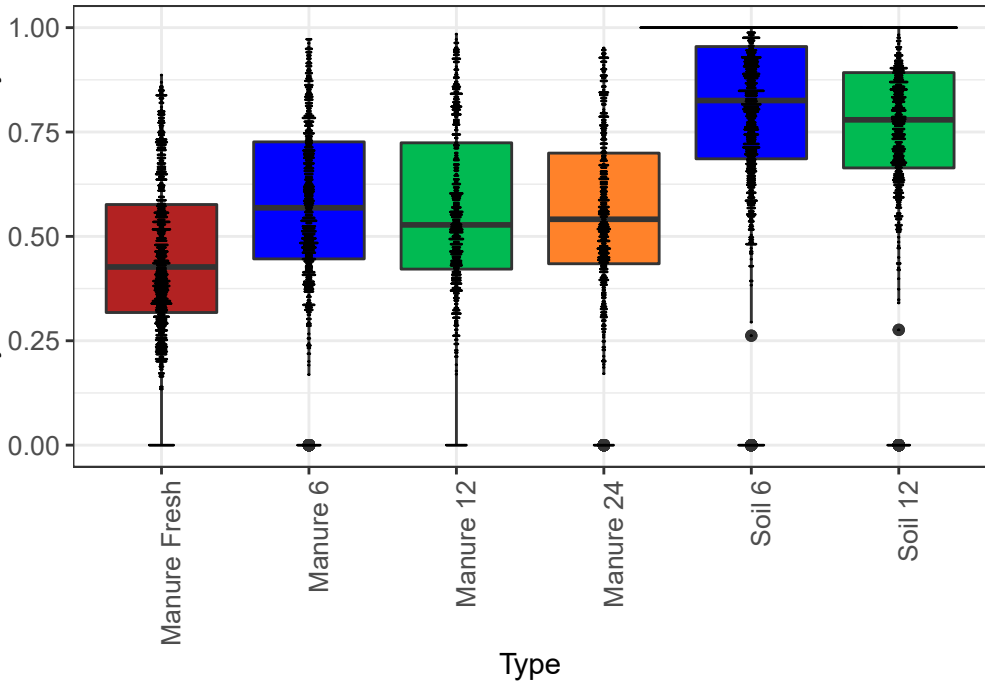

Supplement: FIG S6 [file mbio.00798-21-sf006.pdf]

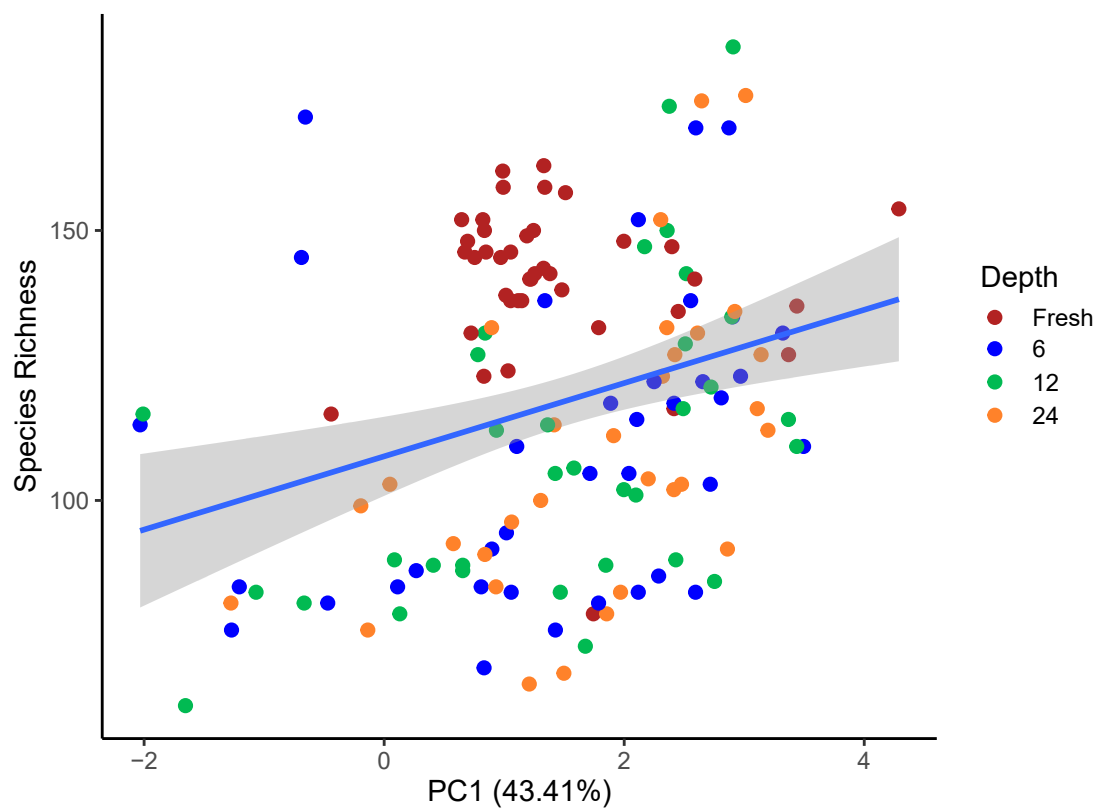

Supplement: FIG S7 [file mbio.00798-21-sf007.pdf]

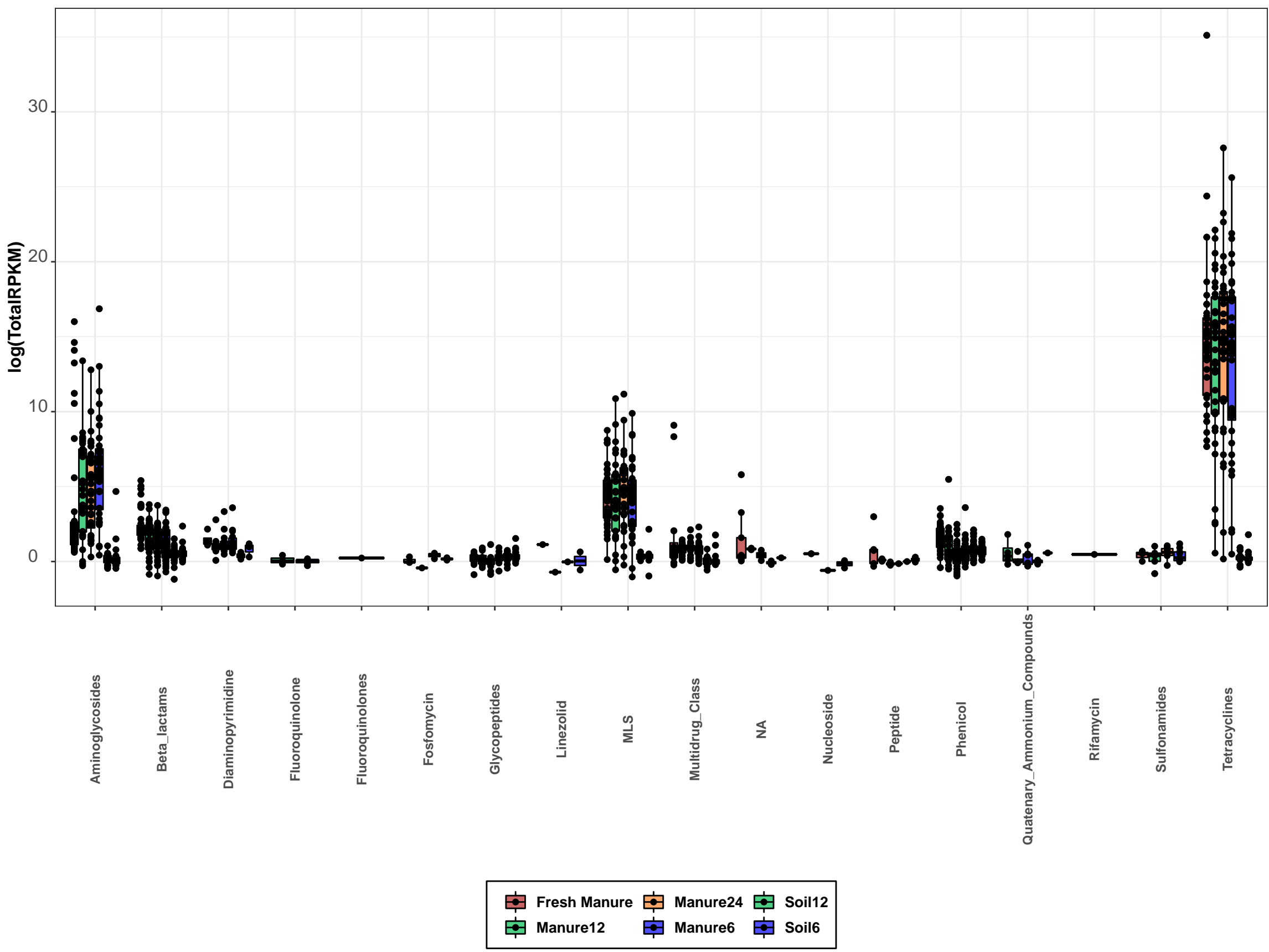

Supplement: FIG S8 [file mbio.00798-21-sf008.pdf]
